# Nutritional Health Knowledge and Literacy among Pregnant Women in the Czech Republic: Analytical Cross-Sectional Study

**DOI:** 10.3390/ijerph20053931

**Published:** 2023-02-22

**Authors:** Klára Papežová, Zlata Kapounová, Veronika Zelenková, Abanoub Riad

**Affiliations:** 1 Department of Public Health, Faculty of Medicine, Masaryk University, 625 00 Brno, Czech Republic; 2 Department of Social Medicine, Department of Social and Assesment Medicine, Charles University, 323 00 Pilsen, Czech Republic; 3 Czech National Centre for Evidence-Based Healthcare and Knowledge Translation (Cochrane Czech Republic, Czech EBHC: JBI Centre of Excellence, Masaryk University GRADE Centre), Institute of Biostatistics and Analyses, Faculty of Medicine, Masaryk University, 625 00 Brno, Czech Republic

**Keywords:** cross-sectional studies, Czech Republic, health literacy, nutrition surveys, pregnancy

## Abstract

Adequate nutrition and the nutritional status of pregnant women are critical for the health of both the mother and the developing foetus. Research has shown a significant impact of nutrition on the child’s health and the future risk of developing chronic noncommunicable diseases (NCDs), such as obesity, diabetes, hypertension, and cardiovascular disease. There is currently no data on the level of nutritional knowledge of Czech pregnant women. This survey aimed to evaluate their level of nutritional knowledge and literacy. An analytical cross-sectional study was conducted in two healthcare facilities in Prague and Pilsen between April and June 2022. An anonymous self-administered paper-form questionnaire for assessing the level of nutritional knowledge (40 items) and the Likert scale for assessing nutrition literacy (5 items) were used. A total number of 401 women completed the questionnaire. An individual’s nutritional knowledge score was calculated and compared with demographic and anamnestic characteristics using statistical methods. The results showed that only 5% of women achieved an overall nutritional score of 80% or more. University education (*p* < 0.001), living in the capital city (*p* < 0.001), experiencing first pregnancy (*p* = 0.041), having normal weight and being overweight (*p* = 0.024), and having NCDs (*p* = 0.044) were statistically significantly associated with a higher nutritional knowledge score. The lowest knowledge scores were found in the areas of optimal energy intake, optimal weight gain, and the role of micronutrients in diet during pregnancy. In conclusion, the study shows limited nutrition knowledge of Czech pregnant women in some areas of nutrition. Increasing nutritional knowledge and nutrition literacy in Czech pregnant women is crucial for supporting their optimal course of pregnancy and the future health of their offspring.

## 1. Introduction

The number of people suffering from overweight, obesity and related diseases is increasing worldwide. According to the World Health Organization (WHO), chronic noncommunicable diseases (NCDs) are the leading cause of morbidity and mortality worldwide. By 2022, 41 million people will die from these causes each year, accounting for 74% of all deaths worldwide. Therefore, great emphasis is placed on early prevention of these NCDs, e.g., hypertension, overweight and obesity, hyperglycemia, and hyperlipidemia [1]. Over time, however, it has become apparent that several diseases may have their origins in an individual’s intrauterine development, and experts have pointed out that maternal lifestyle during pregnancy is linked to serious health consequences and diseases in the child that may develop later in life [2,3,4,5,6,7,8,9]. This concept of ‘nutritional programming’, based on the theory of exposure to specific conditions and lifestyle factors during pregnancy that can determinate an individual’s health later in life, has become accepted dictum [2,3,4,5,6,7,8].

A healthy lifestyle, including a balanced diet and weight control, is undoubtedly crucial for the adequate development of pregnancy in terms of maternal and fetal health [2,4,10,11]. Despite this well-known fact, suboptimal maternal nutrition and weight gain has been seen more often over the past few decades [12]. Women’s pregnancies are increasingly complicated by extreme or morbid obesity, with all its consequences for maternal and fetal health, and despite concerted public health efforts, the proportion of overweight pregnant women continues to rise [13,14].

In today’s world, the online environment offers countless possibilities for obtaining information. With the internet being more accessible to an increasing number of people, it could be thus assumed that easy access to information allows women to obtain all the necessary knowledge related to adequate nutrition, elimination of food risks, and nutrition-related complications associated with pregnancy. However, studies show that this is far from the case and that women do not follow nutritional recommendations [15,16,17,18]. 

A woman’s eating behaviour during pregnancy is influenced by many multifaceted and complex factors. One of the most important factors is the level of nutritional knowledge, the lack of which can be a barrier to adopting healthy behaviours and other postnatal weight management practices. Nutrition knowledge is not only about facts and processes but also about how to apply them in practice [19]. Most studies have documented that the behaviour of pregnant women varies according to, for example, their level of education, age, BMI, number of pregnancies, and sociocultural factors [20,21,22,23]. The influence of socioeconomic factors is also often discussed, such as lower net household income, average educational attainment, and availability of health insurance [24]. 

Lee et al. noted that there is a lack of published research on the assessment of pregnant women’s comprehensive nutrition knowledge [23]. Generally, most previous studies monitored the level of nutritional knowledge around the intake of specific nutrients, such as folic acid [25,26], optimal weight gain [13], iodine intake [27], and fruit and vegetable intake [28], but only a few dealt with comprehensive knowledge [20,23]. One study even addressed areas of nutrition within the context of lifestyle factors of pregnant women [24]. Several studies showed that non-adherence to pregnancy-specific nutritional recommendations was associated with lower levels of nutritional knowledge [20,22,23,29] and indicated that nutrition education during pregnancy was associated with positive pregnancy outcomes [20]. Another study documented that recommendations are often insufficient unless accompanied by support (e.g., through nutrition counselling) to achieve optimal and healthy eating [22]. Many women expect to get all the information they need from a private gynaecologist. Obtaining this type of care has been shown to be a particularly effective method of prevention [24]. Despite this, healthcare providers are not routinely prepared to help pregnant women make informed decisions, and nutritional care is often lacking in primary care for pregnant women [20,30].

There is no definition of the minimum nutritional knowledge that pregnant women should know. The abovementioned studies focusing on the nutritional knowledge of pregnant women are very heterogeneous in this regard, making it difficult to establish a single tool and score system. Women should demonstrate a general overview of all areas of dietary recommendations (which are often country-specific according to national dietary recommendations) without favouring any one area. Each recommendation has a particular rationale related to the health of the woman and the developing foetus. The nutrition knowledge classification is an indicator of success that provides an overall picture; however, far more important is to identify areas in which the level of nutrition knowledge is lowest [12,19].

Nutritional knowledge is one of the cornerstones of health and nutrition literacy, which represents the ability to obtain, understand, and use information that ultimately leads to an increase in one’s own influence on the quality of one’s health. Nutritional knowledge alone does not completely influence an individual’s behaviour, but it can significantly shape their attitudes, which can be reflected in a person’s actions [20].

As for the health and nutritional policy in the Czech Republic on pregnant women regarding nutrition advice and nutritional supplement recommendation during medical appointments, there is not anything like that. For these reasons, it is not entirely clear who should provide this care and counselling to pregnant women, so it is fragmented among health professionals. Although gynaecologists are at the frontline of basic prenatal care, the number of patients and the lack of dedicated time for them detract from prevention, including both nutritional education and the recommendation of dietary supplements [31]. Other competent healthcare professionals in this respect are trained dietitians, of whom there is also a critical shortage and low awareness of their existence in the Czech Republic [32].

In the Czech Republic, no data are available on pregnant women’s nutritional knowledge. Insufficient attention is paid to this issue, and there is a lack of current nutritional recommendations at the national level, and it is not obvious to what extent healthcare professionals should devote time to nutrition education. Thus, women primarily depend on available sources of information (e.g., internet blogs and forums). To increase awareness of this issue, this study aimed to describe the nutritional knowledge level of Czech pregnant women with attention to the influence of selected sociodemographic and anamnestic factors that may contribute to the level of this knowledge and nutrition literacy.

## 2. Materials and Methods

### 2.1. Design and Settings

This analytical cross-sectional survey-based study was conducted between April and June 2022. The questionnaires were distributed to the target participants in a paper form, which they completed on the spot and then dropped in a box. There was no time limit for completion, and the average completion time was 20–30 min. Fully anonymised completed questionnaires were collected securely before being transferred to electronic format.

### 2.2. Participation

The target group was Czech pregnant women. Inclusion criteria were as follows: Czech citizenship, last month of the third trimester (≥36th week of pregnancy), and singleton pregnancy. Exclusion criteria included: non-Czech nationality, low gestational age (˂38 weeks of pregnancy), multiple pregnancies, and age < 18 years.

Data collection occurred in the Gynaecology and Obstetrics Clinic in Pilsen and the Institute for Maternal and Pediatric Care in Prague. These medical facilities were randomly selected; however, there is a significant difference between Prague and Pilsen. Prague is the capital of the Czech Republic (1.3 million inhabitants), and Pilsen represents a smaller town (with 169,000 inhabitants) in the western part of the country. The purpose of the visits to these medical facilities was regular last prenatal check-ups before they come under the control of the birthing facility. Women were asked to participate in the study when they visited an antenatal clinic. Trained health professionals provided women with information about the study, which was also given in the written form.

The minimum sample size required for this study was estimated using Epi Info^TM^ version 7.2.5 (CDC, Atlanta, GA, USA, 2021) utilising the following assumptions [33]:

i. Confidence level (CI) = 95%;
ii. Acceptable error margin = 5%;
iii. Target population size ≈ 111,425 (the average number of live births in the Czech Republic between 2010 and 2021);
iv. Number of clusters = 2;
v. Expected frequency of the primary outcome, which is the satisfactory level of knowledge score > 80%.

At least 384 valid responses were required to establish statistically robust inferences between putative demographic and anamnestic predictors and the current levels of nutritional knowledge. A total of 457 questionnaires were completed, of which 31 were discarded because of incomplete demographic or anamnestic data, and 25 because of the woman’s low gestational age (<38 weeks of pregnancy). Only fully completed questionnaires were used to assess nutritional knowledge (*n* = 401).

### 2.3. Instrument

This study is the first of its kind in the Czech Republic. Thus, it was not possible to use any existing validated measurement. For this purpose, an in-house tool was developed to test women’s nutritional knowledge in multiple areas of nutrition recommendations for pregnancy. The development of the original measurement tool (questionnaire) was thus preceded by an extensive search of scientific literature related to the topic, the selected target group, and the questionnaire methodology. The selection of studies was gradually narrowed down, and the instrument construction was based on modified questionnaires according to selected studies [20,23]. The questions were designed to reflect the national dietary recommendations and, at the same time to test women’s knowledge of several aspects of nutrition, including their ability to understand the meaning of recommendations and to apply them in practice. The accuracy and terminological correctness of the questions were checked and modified in collaboration with dietitians from the Faculty of Medicine at Masaryk University in Brno.

The resulting questionnaire was pilot tested on a sample of 30 women representing the target population and modified based on the feedback as necessary. After final modifications, the reliability of the questionnaire was tested, and the kappa value for test–retest reliability was calculated. The mean value of 0.914 showed perfect test–retest reliability.

The questionnaire consisted of two parts. The first part focused on basic sociodemographic and anamnestic data such as age, level of education, place of residence, pregnancy order, body mass index (BMI), chronic diseases, medication and supplements, adherence to an alternative diet and anthropometric measurements, specifically pregestational height and weight, which were taken from the medical records of each pregnant woman. The cutoff point of age groups (≤28 and 28 years) was determined according to the average age of the first-time mother, which is 28 years in the Czech Republic. 

The second part of the questionnaire consisted of 40 multi-choice questions testing nutritional knowledge divided into five categories. The first category focused on knowledge of micronutrients (iron, calcium, iodine, folic acid, vitamin A, vitamin D, and omega-3 unsaturated fatty acids), the second category focused on knowledge of macronutrients, and the third category on knowledge of nutritional recommendations (e.g., consumption of fruits and vegetables, fish, salt, and fibre) and optimal daily energy intake, weight gain, and the effect of excess weight on a woman’s health. The fourth and fifth categories were devoted to food supplements and food safety concerns (e.g., mercury intake in pregnancy and the risks related to *Listeria monocytogenes* and *Salmonella*).

### 2.4. Outcomes

The nutrition knowledge test (NKT) consisted of 40 items with one correct answer; therefore, the items were considered binary (correct = 1/incorrect = 0). Each item had a ‘do not know’ option to eliminate guessing. The sum of the correct answers (maximum 40) represented the final nutrition knowledge score, which was assessed in relation to the main variables (age, education, BMI, pregnancy order, and presence of disease). A satisfactory level of nutritional knowledge was defined to be >80%.

### 2.5. Ethics

The study was approved by the Ethics Committee of the Faculty of Medicine, Masaryk University, Brno (ref. no. 4/2022), and by the Ethics Committee of the University Hospital in Pilsen and the Faculty of Medicine, University of Pilsen (ref. no. 74/2022), and the Ethics Committee of the Institute for Mother and Child Care in Prague (ref. no. 1/1/2022). The respondents confirmed their consent to participate in the study before completing and submitting a questionnaire.

### 2.6. Analyses

All statistical analyses were carried out using the Statistical Package for Social Sciences (SPSS) version 28.0 (SPSS Inc., Chicago, IL, USA, 2022) and the R-based open software jamovi [34,35]. Initially, the normality of numerical variables, e.g., age, BMI, and knowledge score, was tested using the Shapiro–Wilk test with a significance level (*Sig*.) of <0.05. Consequently, descriptive statistics were used to summarise the sample characteristics (independent variables) and nutritional health knowledge and literacy (dependent variables). Qualitative variables such as education level, pregnancy order, and city were summarised using frequencies and percentages; while numerical variables such as knowledge score were summarised using means and standard deviations (µ ± SD). Then, inferential statistics were carried out to perform hypothesis testing using the chi-squared test (χ^2^), Fisher’s exact test, Mann–Whitney test (U), and Kruskal–Wallis test (H). All analytical tests were carried out with a significance level (*Sig*.) of <0.05.

## 3. Results

### 3.1. Demographic and Ananmnestic Characteristics

Of the 401 questionnaires collected, 264 (65.8%) were collected in Prague and 137 (34.2%) in Pilsen. Most women were aged 30–34 years (40%). The average age of women was 31.6 years, and the median age was 31 years. All of the women were in the third trimester of pregnancy. More than half of the participants had a university degree (57.4%), more of them in Prague (44.9%) than in Pilsen (12.5%). Fifty-three percent of the women were expecting their first child, and their median age was 30.3 years. Most women had a normal body weight at the beginning of pregnancy (63.7%).

The most commonly reported disease accompanying pregnancy was thyroid disease in 14.5% of women, 6% of women reported gestational diabetes mellitus, and 1% of women reported high blood pressure. About a quarter (24.9%) of women were taking multivitamin preparations, 23.7% of women took iron, 14% magnesium and 10.7% folic acid supplements. However, multivitamin supplements are also likely to contain folic acid and iron; contents of these were not controlled in this work due to a huge variability in multivitamin preparations. Only 2.5% of the women reported adherence to an alternative diet that was mostly vegetarian or vegan (Table 1). 

### 3.2. Nutritional Knowledge Items

The test of the level of nutritional knowledge of pregnant women showed limited knowledge of nutrition. Only 5% of women achieved a level of nutritional knowledge higher than 80%. Ten questions with the highest error rate were: a question about the mercury content in fish, a question about the promotion of iron absorption in the diet, a question focused on the energy content of food, a question about vitamin A intake during pregnancy, knowledge about vitamin D intake, recommendations for optimal weight gain, plant sources of calcium, the recommended daily intake of folic acid in pregnancy, the reason for the need for iodine in pregnancy, and the reason for folic acid intake in pregnancy. Among the items where women scored best were sources of folic acid, essential sources of iodine, recommended frequency of fish consumption, the importance of calcium in the diet of a pregnant woman, starting to take folic acid, sources of calcium, foods with high-fat content, the risk of contamination of the diet with *Salmonella*, sources of protein in nutrition, sources of ω − 3 unsaturated fatty acids (Supplementary Table S1).

### 3.3. Nutritional Knowledge Items by Education Level and Age Group

The analysis of NKT showed that the level of nutritional knowledge in pregnant women was highly dependent on the achieved education level. The pregnant women with a university education level (undergraduate and postgraduate degrees) demonstrated better results in questions related to iron (q. #1 and #4), folic acid (q. #6 and #7), omega-3 unsaturated fatty acids (q. #11), vitamin D (q. #14), iodine (q. #15 and #16), vitamin A (q. #17), major nutrients (q. #19 and #20), nutritional recommendations (q. #21–23, #25–26, #28–29, #31–32, and #34), nutritional supplements (q. #35), and food safety (q. #37) knowledge as compared with pregnant women with pre-university educational level (Supplementary Table S2).

Notably, pregnant women with a pre-university education level were more successful with the question about salt consumption in pregnant women (q. #24) compared with pregnant women with a university education level. For further analysis, the effect of the age group was also evaluated. As the average age of first-time mothers in the Czech Republic is 28 years, the pregnant women involved in this study were separated into two groups: (1) ≤28 years old and (2) >28 years old women [36].

The evaluation of the questionnaire showed only a limited effect of age on performance in the NKT. The statistical analysis revealed that pregnant women with higher average age (>28 years old) demonstrated better results in questions related to vitamin D (q. #13), iodine (q. #15 and #16), nutritional recommendations (q. #21 and #28), and nutritional supplements (q. #35) as compared to pregnant women with an average age of 28 years or less; however, the group of ≤28 years-old pregnant women showed a higher percentage of successful answers in the question about salt consumption in pregnant women (q. #24) Table 2.

### 3.4. Nutritional Knowledge Items by BMI and Pregnancy Order

Next, the effect of body mass index (BMI) and pregnancy order on the performance in the NKT was evaluated. It was found that pregnant women scored as underweight or extremely obese demonstrated worse nutritional knowledge in questions related to iron (q. #3 and #4), omega-3 unsaturated fatty acids (q. #12), iodine (q. #15), major nutrients (q. #19 and #20), and nutritional recommendations (q. #30) as compared with pregnant women scored as normal weight, overweight, or obese. For pregnancy order, the pregnant women tested in this study were separated into two groups: (1) primiparous and (2) multiparous. Primiparous women demonstrated a higher percentage of successful answers in questions related to calcium (q. #9), vitamin A (q. #17), nutritional recommendations (q. #22), and food safety (q. #37, #38, and #39) compared with multiparous women. On the other hand, multiparous women demonstrated better results in questions related to iron (q. #3) and vitamin D (q. #14) than primiparous women (Table 3).

### 3.5. Nutritional Knowledge Scores

The nutritional knowledge score (total score) analysis revealed several main factors that influenced the NKT outcome. It was found that one of the most important factors in nutritional knowledge was the education level of pregnant women. Women with a university level of education (undergraduate and graduate degrees) demonstrated better results than those with a pre-university education level. Further, a significant effect was also found in the city of origin. Pregnant women in Prague demonstrated better performance compared with women from Pilsen. Additionally, pregnancy order was also found as a significant factor in the NKT outcome. Primiparous pregnant women showed higher total nutritional knowledge scores than multiparous women. The Kruskal–Wallis test also revealed a significant effect of BMI on the total NKT score. The following analysis showed that the pregnant women with extreme values of BMI (underweight or extremely obese) showed lower scores in NKT compared with pregnant women who scored as normal weight, overweight, or obese. Notably, pregnant women with NCDs demonstrated better results in the NKT than pregnant women without NCDs (Table 4).

A linear regression model was established for the overall nutritional health knowledge score incorporating all the independent variables that were found to be significant in the univariate analyses. According to the model, education level had an adjusted odds ratio (aOR) of 3.06 (CI 95%: 1.97–4.15) and noncommunicable diseases had an aOR of 1.13 (0.03–2.23). On the other hand, city and pregnancy order were not found to be statistically significant in this model (Table 5).

### 3.6. Nutritional Health Literacy

Analysing the results of nutritional health literacy items revealed that education level was the most prominent factor. On comparing the pre-university vs. university groups, differences were statistically significant in information appraisal (*p* = 0.036), utilisation (*p* = 0.031), and help-seeking (*p* = 0.036). For university-educated women, it was more difficult to recognise valuable sources of information, but after receiving the proper information, it was much easier for them to use it. Interestingly, both groups (pre-university and university) reported that it was mainly easy and very easy for them to seek professional help when needed (Table 6).

### 3.7. Determinants of Nutritional Health Literacy

In Table 7, we can observe the subjective assessment of women’s ability to obtain the necessary information, understand it, assess its meaning, use it, and seek professional help in correlation with the main variables. Statistically significant results can be observed for the order of pregnancy, where women who were expecting their first child, despite having higher nutritional knowledge, reported that it was more difficult for them to find the necessary information (information acquisition), understand and appraise it. Similarly, university-educated women with more nutritional knowledge reported that it was more difficult for them to appraise information, use it, and seek professional help than lower-educated women.

## 4. Discussion

The results of the study showed a low level of nutritional knowledge among Czech pregnant women, with only 5% of women achieving more than 80% of correct answers. These results could be comparable to a study where Lee et al. reported very similar overall nutritional knowledge of pregnant women. Out of a group of 114 women, only 2% demonstrated a level of nutrition knowledge during pregnancy higher than 80% [23]. The results are therefore very similar to those of our study. The next level of assessment of nutrition knowledge is problematic because there is no cutoff score that clearly delineates the boundaries of each grade/level of nutrition knowledge. For this reason, studies usually use similar scores to allow comparison of results [12].

Tests based on a total score usually require the use of proven methods. When assessing nutritional knowledge, it is more meaningful to assess individual areas, as good knowledge may be recorded in some, while bad knowledge may be noted in others [37]. For this reason, and in line with other studies, our evaluation focused on the error rate of questions with the lowest nutritional scores. Focusing on questions with the highest error rates can help to identify areas where preventive interventions need to be strengthened.

In the studied population of pregnant women, the lowest knowledge was demonstrated in the question asking about the types of fish with mercury content: 84% of women answered incorrectly. At the same time, 87.8% of women correctly answered the question, “*How many times a week it is recommended for pregnant women to eat fish?*”. The connection between these two questions may point to the difficulty of using knowledge in practice. Lee et al. also found women had limited knowledge of risky foods containing mercury [23]. The second question with the highest error rate was related to the promotion of dietary iron absorption. During pregnancy, the need for iron is many times higher than the need for other micronutrients [13,30]; 74.3% of women did not know that vitamin C supports the absorption of iron from the diet. The questions on iron absorption were asked primarily to test the level of nutritional knowledge in women at risk of low iron intake, which includes those on alternative diets (vegetarianism, veganism). Due to the low number of these women in the study population (2.5%), it was not possible to evaluate this fact. However, the results surprisingly showed the absence of this knowledge in the majority of pregnant women. This may be due to a lack of awareness of the increased need for iron in pregnancy with little or no interest in this issue, even though at least 23.7% of women supplemented with iron.

Knowledge of daily energy needs is very important for pregnant women, which is one of the prerequisites for weight management; 73.8% of women did not answer correctly which of the presented dishes contained 1500 kJ and 56.4% of women did not know by how many kJ the daily energy intake increased in the second and third trimesters. Additionally, 66.3% of women did not correctly state the optimal weight gain during pregnancy for women who had a normal body weight at conception. Downs et al. also concluded in their study that women did not have the necessary knowledge about the recommended weight gain during pregnancy [13]. Shub et al. also confirmed in a study of 364 pregnant women that women had limited knowledge about weight gain during pregnancy [38]. A systematic review and meta-synthesis of qualitative research gathering evidence on the understanding, perception, and evaluation of women’s optimal weight gain during pregnancy reported that women were not aware of optimal weight gains during pregnancy [14].

The question about vitamin A and its need during pregnancy was difficult for most women to answer. When asked whether its need is increased or decreased in pregnancy, 70% of women answered incorrectly. Similarly, low knowledge was evident for vitamin D. In this study, 66.3% of women answered incorrectly on the importance of increased need in pregnancy; 50.9% of women could not even identify the source of vitamin D in their diet. Other studies on knowledge about vitamin D showed that 78.5% of women presented a good knowledge [20].

Another very interesting finding was on the use of folic acid during pregnancy. Even though 88.8% of women knew when to start taking folic acid, 60.8% of women did not know the recommended daily intake and 56.6% of women did not know the reason for taking folic acid in pregnancy. The same difficulty was encountered in a study of 150 women in New South Wales, Australia, which found that most women in the study population did not know the reason for taking folic acid or the adequate recommended daily intake of folic acid and iodine from food and supplements during pregnancy [39]. Different results were found in an Iranian cross-sectional study of 265 women, which found good knowledge of folic acid use during pregnancy and that only 34.1% of women expressed negative attitudes towards its use [26]. Other similar studies also demonstrate the lack of nutritional knowledge of pregnant women regarding folic acid intake [25]. 

The results of the assessment of nutritional knowledge showed the absence of knowledge of pregnant women in important areas of nutritional recommendations. It is important for pregnant women to strengthen their knowledge on recommended energy intake during pregnancy, optimal weight gain, and in terms of micronutrients, especially knowledge about the use of folic acid. The results indicate difficulties with using information in practice, and women often did not know the reason for specific nutritional recommendations.

The factor of level of education proved to be a very strong factor related to the level of nutritional knowledge, where university-educated women had a higher level of nutritional knowledge than other women with a lower level of education. In our sample, 57.4% of women were university educated, which could have influenced the results due to the low proportion of women with the lowest education in the sample. The bigger proportion of university-educated women in this study could be explained by their higher willingness to fill in the questionnaire. For the investigation, we also chose large cities where universities are represented, and it could be thus assumed many educated women would live there.

The group of women was divided into two groups according to the age of first-time mothers, which in the Czech Republic is 28 years. The difference between the level of nutritional knowledge of women aged ≤ 28 years and older women was investigated. The reason for dividing the group by age was the assumption that first-time mothers may have a higher level of nutritional knowledge. The results, however, showed a higher level of nutritional knowledge of women older than 28 years, but it was not statistically significant. The assumption of a higher level of nutritional knowledge according to the national average age of first-time mothers was wrong. In our group of women, the average age of first-time mothers was 30.4 years, which may be related to the high proportion of university-educated women who have children at a later age than the national average. Regarding women’s age, these results are consistent with the studies mentioned above, where younger women demonstrated lower levels of nutritional knowledge.

Women’s weight at the beginning of pregnancy has become the subject of many expert discussions and is very important for the health of both the woman and the foetus. If a woman’s weight at conception is not optimal, the woman must be aware of the possible risks and, above all, the energy value of the diet and optimal weight gain [11,40].

Statistical relationships between the level of nutritional knowledge and women’s weight at the beginning of pregnancy have been demonstrated. Underweight and extremely obese women showed lower knowledge than other women. The problem is found in the lower level of education of these risk groups in the issue of recommending energy intake in the second and third trimesters (*p* = 0.014). In this regard, it is desirable that especially at risk groups of women have sufficient knowledge about the optimal increase in energy requirements for proper weight gain during pregnancy.

In a survey of Scottish women were pregnant women recruited from a cohort study of severely obese pregnant women. Severely obese pregnant women in this study also had lower scores on general nutrition knowledge than the group with normal weight. The results remained significant after controlling for education level [41]. Education should be aimed especially at women who do not have a normal body weight at the beginning of pregnancy and they should be consistently educated about appropriate energy intake and optimal weight gain during pregnancy.

Our results showed a higher level of nutritional knowledge of first-time mothers compared to that of women with more children. It was hypothesised that there might be a significant relationship between pregnancy order and level of nutritional knowledge. These women may have more time to educate themselves or perceive a higher degree of responsibility for the optimal course of pregnancy. Indeed, primiparous women demonstrated a higher level of knowledge in the area of food safety (mercury content in fish (*p* = 0.011), risks associated with *Listeria monocytogenes* (*p* = 0.003) and risks of this bacterium to the foetus (*p* = 0.01). This assumption thus may be correct.

A cross-sectional study from Ghana found satisfactory knowledge of food risks among pregnant women but suggested that food safety knowledge may not be associated with appropriate nutritional behaviour [42]. In this study, the following factors were observed in relation to the level of nutritional knowledge of pregnant women: age, level of education, city, pregnancy order, body mass index (BMI), noncommunicable diseases (NCDs), medications and supplements, and alternative diet. Statistical significance was found for the following factors: education (*p* < 0.001), city (*p* < 0.001), pregnancy order (*p* = 0.041), BMI (*p* = 0.024), and NCDs (*p* = 0.044). 

Our study demonstrated a higher nutritional knowledge score among women with higher education compared to other groups of women. The factor of the place of data collection is debatable, as it was a question of large cities with a high proportion of university-educated respondents, and the connection of this result is not entirely clear. According to the national survey from 2021, the population level of university education was 18.7%, the highest concentration was in Prague (35.9%), and lower percentage was in Pilsen (14.5%) [43]. The sample presented included a higher percentage of university- educated people overall (57.4%), of which 44.9% were in Prague and 12.5% in Pilsen. The first pregnancy was found to be significant when women demonstrated a higher level of nutritional knowledge. Very interesting was the finding on BMI, where women with underweight and extreme obesity turned out to be very risky groups. These women should be given significant care as part of prevention, as there is a risk of harming the health of both the woman and the child from incorrect nutritional behaviour during pregnancy. The presence of disease was also found to be a statistically significant factor. Here we can assume a higher level of education of women by health professionals due to the existence of health problems, as well as a possibly higher interest of women in their health and the health of their children [44].

In the field of research, we do not find many studies to compare with our results. We can compare the results from the Istanbul study on a sample of 736 pregnant women, which showed a relationship between the age of the women and the level of nutritional knowledge. Women aged < 18 years presented the lowest level of knowledge compared to the other age groups, 25–29 years, 30–34 years, and ≥35 years. In this study, high school graduates had higher scores of nutritional knowledge than primary school graduates, and finally, the order of pregnancy emerged as significant, with women in their first pregnancy having higher nutritional knowledge scores than those with more than five pregnancies. BMI was not a statistically significant factor in this study [18]. The results are consistent with our study when considering the factors of age, education, and pregnancy order. Unlike in this study, BMI was proven to be a statistically significant factor in our study.

In contrast, a cross-sectional study assessing the nutritional knowledge of pregnant women according to the Australian Dietary Guidelines confirmed significant demographic differences in nutritional knowledge scores. Multiple regression analysis confirmed significant independent effects of education level, income, age, stage of pregnancy, language, and health/nutrition qualifications on respondents’ nutritional knowledge scores [12]. 

Some studies focus on isolated knowledge of nutrition in conjunction with sociodemographic data of pregnant women. A Turkish study investigated the knowledge of iodine in 150 pregnant women aged 19 to 45 in relation to the sociodemographic characteristics of the study participants. Only 68% of women knew that iodine deficiency could have serious consequences during pregnancy. Knowledge was significantly associated with the level of education (*p* < 0.001), but women’s age, trimester, and parity were statistically insignificant [45].

Assessing the influence of sociodemographics and other factors on nutritional knowledge is very important from the point of view of searching for risk groups of pregnant women on whom preventive and intervention strategies should focus. 

This study assessed the ability of women with higher education and women with less education to obtain, use, and recognise valuable information, and the difficulty of seeking professional help. Responses were compared between women with a university and non-university education. Only statistically significant items were assessed. These abilities correspond to health literacy, and nutritional knowledge is one of the cornerstones of health literacy, which represents the ability to acquire, understand, and use information that ultimately leads to an increase in one’s own influence on the quality of health. Nutritional knowledge itself will not completely influence an individual’s behaviour, but it can significantly shape their attitudes, which can be reflected in a person’s actions [46].

From the results, we can conclude that women who demonstrated a higher level of knowledge in the study could think more about where to find valuable information, think more about its use in practice, etc., compared to women who demonstrated a lower level of knowledge.

### 4.1. Limitations

A limitation of the study was the inability to ensure an equal distribution of the group in terms of education. The tertiary level of education predominated, which limits the generalisation of the results. This can be explained by the greater interest of women with higher education in this issue and, thus, their willingness to complete the questionnaire. A limit was also maternity hospitals in large cities, where there may be a higher concentration of university-educated women. Another limitation could be the questionnaire’s scope and the questions’ difficulty. Another limitation may have been guessing responses, although each question was provided with a “do not know” option.

### 4.2. Strengths

This study is the first to be conducted in the Czech Republic. So far, this topic has not been addressed. The harvested sample has an optimal size and decent representativeness. The selection of a group of women in the third trimester of pregnancy reflected all the knowledge gained during pregnancy. This heterogeneous sample allowed for subgroup analysis across education level, BMI, and pregnancy order, among other factors. Finally, the current study highlighted the problem of nutritional knowledge of pregnant women and the absence and limited availability of expert and evidence-based recommendations. We appeal to all interested parties to continue to address this situation, as this issue is currently greatly underestimated in the Czech Republic.

### 4.3. Implications

The results of this study point to the need to appeal to professionals and politicians to raise awareness about the need for changes that can lead to an increase in the level of nutritional knowledge of pregnant women. This issue appears to be severely underestimated at present because of the potential impact on pregnant woman’s health and children’s health in the future. So, in the future, it is essential to focus on constantly improving the level of nutritional knowledge and nutritional literacy. One of the objectives of national health policy should be nationwide primary prevention, which should already be aimed at the young generation, as prenatal interventions often come at a time when they are not effective to a sufficient degree. Pre-conception care and preparation for parenthood should be an obvious part of not only health care but also the education system in any developed society. Attention needs to be focused on increasing the level of education of pregnant women on nutritional recommendations and making them available, for example, through the portals of the Ministry of Health or professional societies. Awareness of the importance of dietary recommendations should be strengthened by developing effective campaigns and national programs aimed at the target group of women. Healthcare providers should be the first and reliable source of nutritional information, interprofessional cooperation should be strengthened, and free care by dietitians should be increased. Last but not least, regular measurement and evaluation of the level of nutritional knowledge of pregnant women should be ensured and a validated tool should be developed at national level for this regular measurement and evaluation. 

The need for regular measurement and evaluation of pregnant women’s nutritional knowledge level must be emphasised and continued. Knowing the level of knowledge is a prerequisite for effective prevention, which includes the development of effective and comprehensible nutrition-based recommendations that can target areas of concern and help in effectively developing campaigns and programs aimed at target groups.

## 5. Conclusions

The study’s results showed low nutritional knowledge among Czech pregnant women: only 5% of women demonstrated knowledge above 80%. Lack of knowledge was demonstrated in key areas such as optimal energy intake, optimal weight gain, and micronutrients. Women found it difficult to understand the meaning of the recommendations and their use in practice. Level of education (*p* < 0.001), city (*p* < 0.001), order of pregnancy (*p* = 0.041), BMI (*p* = 0.024), and NCDs (*p* = 0.044) were statistically significant factors. For targeted prevention, it is necessary to continue to measure and evaluate the level of internal knowledge. Nutritional education should therefore increase the level of knowledge in the areas mentioned above, which may lead to changes in inappropriate eating habits and support the adoption of healthy eating habits.

## Figures and Tables

**Table 1:** Demographic and anamnestic characteristics of pregnant women participating in the nutritional health survey, April–May 2022 (*n* = 401).

Variable	Outcome	Frequency (*n*)	Percentage (*%*)
Age Group	18–28 years old	100	25
	>28 years old	300	75
Education Level	Basic Education	15	3.7
	Secondary School Completed by an Apprenticeship Exam	23	5.7
	High School Completed by a Matriculation Exam	115	28.7
	Higher Professional Education	18	4.5
	University: Undergraduate	226	56.4
	University: Postgraduate	4	1
City	Prague	264	65.8
	Pilsen	137	34.2
Pregnancy Order	First Pregnancy	210	53
	Second Pregnancy	144	36.4
	Third Pregnancy	36	9.1
	Fourth Pregnancy	5	1.3
	≥Fifth Pregnancy	1	3
Body Mass Index (BMI)	Underweight (<18.5)	21	5.4
	Normal Weight (18.5–24.9)	249	63.7
	Overweight (25–29.9)	69	17.6
	Obese (30–34.9)	31	7.9
	Extremely Obese (≥35)	21	5.4
Chronic Disease	Diabetes Mellitus Type I and II	3	0.8
	Gestational Diabetes Mellitus	24	6.0
	Chronic Hypertension	4	1.0
	Thyroid Disease	58	14.5
	Psychologic Disorder	4	1.0
	Other	34	8.5
	Total	127	31.7
Medications and Supplements	Folic Acid Supplements	43	10.7
	Iron Supplements	95	23.7
	Magnesium Supplements	56	14
	Multivitamin Supplements	100	24.9
	Total	271	67.6
Alternative Diet	Vegan	2	0.5
	Vegetarian	7	1.7
	Another Special Diet	1	0.2
	Total	10	2.5

**Table 2:** Nutritional health knowledge items of pregnant women participating in the nutritional health survey according to education level and age group, April–May 2022 (*n* = 401).

No.	Topic	Education Level			Age Group			Total (*n* = 401)
		Pre-University (*n* = 171)	University (*n* = 230)	*Sig.*	≤28 Years Old (*n* = 100)	>28 Years Old (*n* = 300)	*Sig.*	
1	Iron	126 (73.7%)	196 (85.2%)	0.004	79 (79%)	242 (80.7%)	0.717	322 (80.3%)
2		38 (22.2%)	65 (28.3%)	0.171	26 (26%)	77 (25.7%)	0.947	103 (25.7%)
3		85 (49.7%)	124 (53.9%)	0.404	59 (59%)	149 (49.7%)	0.106	209 (52.1%)
4		113 (66.1%)	180 (78.3%)	0.007	68 (68%)	225 (75%)	0.171	293 (73.1%)
5	Folic Acid	146 (85.4%)	191 (83%)	0.527	83 (83%)	253 (84.3%)	0.753	337 (84%)
6		50 (29.2%)	107 (46.5%)	<0.001	34 (34%)	122 (40.7%)	0.237	157 (39.2%)
7		56 (32.7%)	118 (51.3%)	<0.001	38 (38%)	136 (45.3%)	0.200	174 (43.4%)
8	Calcium	151 (88.3%)	211 (91.7%)	0.251	87 (87%)	274 (91.3%)	0.206	362 (90.3%)
9		47 (27.5%)	93 (40.4%)	0.007	31 (31%)	108 (36%)	0.363	140 (34.9%)
10		149 (87.1%)	204 (88.7%)	0.634	88 (88%)	264 (88%)	1.000	353 (88%)
11	ω − 3	152 (88.9%)	221 (96.1%)	0.005	90 (90%)	282 (94%)	0.175	373 (93%)
12		126 (73.7%)	185 (80.4%)	0.109	78 (78%)	232 (77.3%)	0.890	311 (77.6%)
13	Vit. D	82 (48%)	115 (50%)	0.685	38 (38%)	159 (53%)	0.009	197 (49.1%)
14		47 (27.5%)	88 (38.3%)	0.024	29 (29%)	106 (35.3%)	0.246	135 (33.7%)
15	Iodine	138 (80.7%)	206 (89.6%)	0.012	77 (77%)	266 (88.7%)	0.004	344 (85.8%)
16		55 (32.2%)	109 (47.4%)	0.002	32 (32%)	131 (43.7%)	0.040	164 (40.9%)
17	Vit. A	61 (35.7%)	118 (51.3%)	0.002	40 (40%)	139 (46.3%)	0.270	179 (44.6%)
18	Major Ntr.	158 (92.4%)	214 (93%)	0.805	96 (96%)	275 (91.7%)	0.148	372 (92.8%)
19		126 (73.7%)	192 (83.5%)	0.017	82 (82%)	235 (78.3%)	0.434	318 (79.3%)
20		144 (84.2%)	223 (97%)	<0.001	87 (87%)	279 (93%)	0.062	367 (91.5%)
21	Nutritional Recommendations	61 (35.7%)	116 (50.4%)	0.003	34 (34%)	143 (47.7%)	0.017	177 (44.1%)
22		115 (67.3%)	184 (80%)	0.004	74 (74%)	225 (75%)	0.842	299 (74.6%)
23		141 (82.5%)	211 (91.7%)	0.005	87 (87%)	264 (88%)	0.792	352 (87.8%)
24		116 (67.8%)	127 (55.2%)	0.011	72 (72%)	170 (56.7%)	0.007	243 (60.6%)
25		119 (69.6%)	186 (80.9%)	0.009	76 (76%)	228 (76%)	1.000	305 (76.1%)
26		93 (54.4%)	162 (70.4%)	<0.001	67 (67%)	187 (62.3%)	0.401	255 (63.6%)
27		135 (78.9%)	196 (85.2%)	0.102	84 (84%)	246 (82%)	0.649	331 (82.5%)
28		121 (70.8%)	209 (90.9%)	<0.001	67 (67%)	263 (87.7%)	<0.001	330 (82.3%)
29		61 (35.7%)	121 (52.6%)	<0.001	42 (42%)	140 (46.7%)	0.417	182 (45.4%)
30		66 (38.6%)	109 (47.4%)	0.079	45 (45%)	130 (43.3%)	0.771	175 (43.6%)
31		32 (18.7%)	73 (31.7%)	0.003	33 (33%)	72 (24%)	0.076	105 (26.2%)
32		46 (26.9%)	89 (38.7%)	0.013	29 (29%)	106 (35.3%)	0.246	135 (33.7%)
33		79 (46.2%)	106 (46.1%)	0.982	48 (48%)	137 (45.7%)	0.685	185 (46.1%)
34		128 (74.9%)	192 (83.5%)	0.033	79 (79%)	241 (80.3%)	0.773	320 (79.8%)
35	Supp.	142 (83%)	214 (93%)	0.002	83 (83%)	272 (90.7%)	0.036	356 (88.8%)
36		50 (29.2%)	71 (30.9%)	0.725	36 (36%)	84 (28%)	0.131	121 (30.2%)
37	Food Safety	20 (11.7%)	45 (19.6%)	0.034	20 (20%)	45 (15%)	0.240	65 (16.2%)
38		125 (73.1%)	168 (73%)	0.990	68 (68%)	224 (74.7%)	0.193	293 (73.1%)
39		155 (90.6%)	216 (93.9%)	0.218	91 (91%)	279 (93%)	0.511	371 (92.5%)
40		75 (43.9%)	100 (43.5%)	0.939	41 (41%)	134 (44.7%)	0.522	175 (43.6%)

Chi-squared test (χ^2^) and Fisher’s exact test were used with a significance level ≤ 0.05.

**Table 3:** Nutritional health knowledge items of pregnant women participating in the nutritional health survey according to BMI and pregnancy order, April–May 2022 (*n* = 401).

No.	Topic	Body Mass Index (BMI) Level			Pregnancy Order			Total (*n* = 401)
		Underweight and Extremely Obese (*n* = 42)	Normal, Overweight, and Obese (*n* = 349)	*Sig.*	First (*n* = 210)	≥Second (*n* = 186)	*Sig.*	
1	Iron	34 (81%)	279 (79.9%)	0.877	170 (81%)	150 (80.6%)	0.938	322 (80.3%)
2		7 (16.7%)	94 (26.9%)	0.151	61 (29%)	41 (22%)	0.112	103 (25.7%)
3		15 (35.7%)	191 (54.7%)	0.020	120 (57.1%)	85 (45.7%)	0.023	209 (52.1%)
4		22 (52.4%)	264 (75.6%)	0.001	153 (72.9%)	136 (73.1%)	0.953	293 (73.1%)
5	Folic Acid	34 (81%)	297 (85.1%)	0.481	178 (84.8%)	154 (82.8%)	0.596	337 (84%)
6		11 (26.2%)	145 (41.5%)	0.055	92 (43.8%)	64 (34.4%)	0.056	157 (39.2%)
7		15 (35.7%)	154 (44.1%)	0.298	97 (46.2%)	77 (41.4%)	0.338	174 (43.4%)
8	Calcium	38 (90.5%)	314 (90%)	1.000	191 (91%)	166 (89.2%)	0.570	362 (90.3%)
9		12 (28.6%)	128 (36.7%)	0.301	83 (39.5%)	56 (30.1%)	0.050	140 (34.9%)
10		36 (85.7%)	307 (88%)	0.674	182 (86.7%)	167 (89.8%)	0.338	353 (88%)
11	ω − 3	37 (88.1%)	330 (94.6%)	0.099	199 (94.8%)	170 (91.4%)	0.185	373 (93%)
12		27 (64.3%)	277 (79.4%)	0.026	162 (77.1%)	144 (77.4%)	0.948	311 (77.6%)
13	Vit. D	22 (52.4%)	169 (48.4%)	0.628	97 (46.2%)	98 (52.7%)	0.197	197 (49.1%)
14		11 (26.2%)	120 (34.4%)	0.288	54 (25.7%)	81 (43.5%)	<0.001	135 (33.7%)
15	Iodine	31 (73.8%)	305 (87.4%)	0.017	179 (85.2%)	160 (86%)	0.825	344 (85.8%)
16		14 (33.3%)	145 (41.5%)	0.306	83 (39.5%)	80 (43%)	0.482	164 (40.9%)
17	Vit A	16 (38.1%)	160 (45.8%)	0.340	106 (50.5%)	72 (38.7%)	0.019	179 (44.6%)
18	Major Ntr.	38 (90.5%)	326 (93.4%)	0.514	199 (94.8%)	169 (90.9%)	0.131	372 (92.8%)
19		27 (64.3%)	283 (81.1%)	0.011	172 (81.9%)	142 (76.3%)	0.173	318 (79.3%)
20		34 (81%)	325 (93.1%)	0.007	194 (92.4%)	169 (90.9%)	0.585	367 (91.5%)
21	Nutritional Recommendations	16 (38.1%)	158 (45.3%)	0.377	88 (41.9%)	87 (46.8%)	0.330	177 (44.1%)
22		28 (66.7%)	267 (76.5%)	0.162	168 (80%)	130 (69.9%)	0.020	299 (74.6%)
23		39 (92.9%)	306 (87.7%)	0.449	183 (87.1%)	166 (89.2%)	0.518	352 (87.8%)
24		27 (64.3%)	211 (60.5%)	0.631	125 (59.5%)	114 (61.3%)	0.720	243 (60.6%)
25		30 (71.4%)	267 (76.5%)	0.467	155 (73.8%)	147 (79%)	0.223	305 (76.1%)
26		23 (54.8%)	224 (64.2%)	0.232	141 (67.1%)	112 (60.2%)	0.152	255 (63.6%)
27		32 (76.2%)	292 (83.7%)	0.224	177 (84.3%)	151 (81.2%)	0.414	331 (82.5%)
28		32 (76.2%)	290 (83.1%)	0.267	173 (82.4%)	154 (82.8%)	0.914	330 (82.3%)
29		14 (33.3%)	165 (47.3%)	0.087	104 (49.5%)	76 (40.9%)	0.084	182 (45.4%)
30		11 (26.2%)	161 (46.1%)	0.014	98 (46.7%)	76 (40.9%)	0.245	175 (43.6%)
31		8 (19%)	93 (26.6%)	0.288	63 (30%)	42 (22.6%)	0.095	105 (26.2%)
32		10 (23.8%)	121 (34.7%)	0.159	69 (32.9%)	65 (34.9%)	0.661	135 (33.7%)
33		27 (64.3%)	154 (44.1%)	0.013	98 (46.7%)	84 (45.2%)	0.764	185 (46.1%)
34		35 (83.3%)	277 (79.4%)	0.546	168 (80%)	149 (80.1%)	0.979	320 (79.8%)
35	Supp.	35 (83.3%)	312 (89.4%)	0.297	187 (89%)	166 (89.2%)	0.949	356 (88.8%)
36		13 (31%)	103 (29.5%)	0.847	68 (32.4%)	52 (28%)	0.339	121 (30.2%)
37	Food Safety	4 (9.5%)	60 (17.2%)	0.204	42 (20%)	20 (10.8%)	0.011	65 (16.2%)
38		29 (69%)	257 (73.6%)	0.526	167 (79.5%)	123 (66.1%)	0.003	293 (73.1%)
39		38 (90.5%)	325 (93.1%)	0.524	197 (93.8%)	171 (91.9%)	0.468	371 (92.5%)
40		14 (33.3%)	157 (45%)	0.150	105 (50%)	69 (37.1%)	0.010	175 (43.6%)

Chi-squared test (χ^2^) and Fisher’s exact test were used with a significance level ≤ 0.05.

**Table 4:** Nutritional health knowledge scores of pregnant women participating in the nutritional health survey, April–May 2022 (*n* = 401).

Variable	Outcome	Iron (0–4)	Folic Acid (0–3)	Calcium (0–3)	ω-3 (0–2)	Vit. D (0–2)	Iodine (0–2)	Vit. A (0–1)	Major Ntr. (0–3)	Ntr. Rec. (0–14)	Supp. (0–2)	Safety (0–4)	Total (0–40)	*Sig.*
Age	≤28 yo	2.3 ± 1.0	1.6 ± 0.8	2.1 ± 0.7	1.7 ± 0.6	0.7 ± 0.7	1.1 ± 0.7	0.4 ± 0.5	2.7 ± 0.6	8.4 ± 2.5	1.2 ± 0.6	2.2 ± 0.9	24.2 ± 5.1	0.068
	>28 yo	2.3 ± 0.9	1.7 ± 0.9	2.2 ± 0.7	1.7 ± 0.5	0.9 ± 0.8	1.3 ± 0.6	0.5 ± 0.5	2.6 ± 0.7	8.5 ± 2.6	1.2 ± 0.5	2.3 ± 0.9	25.2 ± 5.4	
Education	Pre-Uni	2.1 ± 1.0	1.5 ± 0.8	2.0 ± 0.7	1.6 ± 0.6	0.8 ± 0.7	1.1 ± 0.7	0.4 ± 0.5	2.5 ± 0.7	7.7 ± 2.7	1.1 ± 0.6	2.2 ± 0.9	23.0 ± 5.5	<0.001
	University	2.5 ± 0.9	1.8 ± 0.9	2.2 ± 0.7	1.8 ± 0.5	0.9 ± 0.8	1.4 ± 0.7	0.5 ± 0.5	2.7 ± 0.6	9.1 ± 2.2	1.2 ± 0.5	2.3 ± 0.9	26.3 ± 4.8	
City	Prague	2.4 ± 0.9	1.7 ± 0.9	2.2 ± 0.7	1.7 ± 0.5	0.8 ± 0.8	1.3 ± 0.6	0.5 ± 0.5	2.7 ± 0.6	8.6 ± 2.5	1.2 ± 0.5	2.4 ± 0.9	25.5 ± 5.3	<0.001
	Pilsen	2.2 ± 1.0	1.5 ± 0.9	2.1 ± 0.6	1.7 ± 0.6	0.8 ± 0.7	1.2 ± 0.7	0.3 ± 0.5	2.5 ± 0.7	8.1 ± 2.6	1.2 ± 0.6	2.0 ± 0.8	23.7 ± 5.3	
Pregnancy	First	2.4 ± 1.0	1.8 ± 0.9	2.2 ± 0.6	1.7 ± 0.5	0.7 ± 0.7	1.3 ± 0.7	0.5 ± 0.5	2.7 ± 0.6	8.6 ± 2.5	1.2 ± 0.6	2.4 ± 0.9	25.5 ± 5.1	0.041
	≥Second	2.2 ± 0.9	1.6 ± 0.9	2.1 ± 0.7	1.7 ± 0.6	1.0 ± 0.8	1.3 ± 0.7	0.4 ± 0.5	2.6 ± 0.7	8.4 ± 2.5	1.2 ± 0.6	2.1 ± 0.9	24.4 ± 5.5	
BMI	<18.5	1.7 ± 1.0	1.5 ± 0.8	2.1 ± 0.5	1.5 ± 0.7	0.9 ± 0.8	1.0 ± 0.8	0.4 ± 0.5	2.3 ± 0.7	7.8 ± 3.0	1.2 ± 0.6	2.1 ± 1.0	22.5 ± 6.1	0.024
	18.5–24.9	2.4 ± 0.9	1.7 ± 0.8	2.1 ± 0.7	1.7 ± 0.5	0.8 ± 0.8	1.3 ± 0.7	0.4 ± 0.5	2.7 ± 0.6	8.4 ± 2.5	1.2 ± 0.6	2.2 ± 0.9	25.0 ± 5.1	
	25–29.9	2.4 ± 1.0	1.7 ± 0.9	2.2 ± 0.7	1.8 ± 0.5	0.8 ± 0.8	1.3 ± 0.7	0.6 ± 0.5	2.7 ± 0.6	8.8 ± 2.5	1.1 ± 0.6	2.4 ± 0.9	25.7 ± 5.6	
	30–34.9	2.4 ± 1.0	1.8 ± 0.8	2.2 ± 0.6	1.8 ± 0.4	0.9 ± 0.7	1.2 ± 0.6	0.5 ± 0.5	2.7 ± 0.7	9.2 ± 2.4	1.1 ± 0.5	2.6 ± 0.9	26.3 ± 5.2	
	≥35	2.0 ± 1.0	1.4 ± 0.8	2.0 ± 0.6	1.6 ± 0.7	0.7 ± 0.7	1.1 ± 0.7	0.3 ± 0.5	2.4 ± 0.7	8.0 ± 2.9	1.1 ± 0.7	2.0 ± 0.7	22.6 ± 6.1	
BMI Level	UW and EO	1.9 ± 1.0	1.4 ± 0.8	2.1 ± 0.6	1.5 ± 0.7	0.8 ± 0.7	1.1 ± 0.7	0.4 ± 0.5	2.4 ± 0.7	7.9 ± 2.9	1.1 ± 0.6	2.0 ± 0.8	22.5 ± 6.0	0.005
	N and O	2.4 ± 0.9	1.7 ± 0.9	2.2 ± 0.7	1.7 ± 0.5	0.8 ± 0.8	1.3 ± 0.7	0.5 ± 0.5	2.7 ± 0.6	8.6 ± 2.5	1.2 ± 0.6	2.3 ± 0.9	25.3 ± 5.2	
NCD	No	2.3 ± 0.9	1.6 ± 0.9	2.1 ± 0.7	1.7 ± 0.6	0.8 ± 0.7	1.3 ± 0.7	0.4 ± 0.5	2.6 ± 0.7	8.3 ± 2.6	1.2 ± 0.6	2.2 ± 0.9	24.5 ± 5.5	0.044
	Yes	2.4 ± 0.9	1.7 ± 0.8	2.1 ± 0.7	1.8 ± 0.4	0.9 ± 0.8	1.3 ± 0.6	0.5 ± 0.5	2.7 ± 0.6	8.8 ± 2.3	1.2 ± 0.6	2.3 ± 0.9	25.7 ± 5.1	
Medicines	No	2.2 ± 0.9	1.6 ± 0.9	2.2 ± 0.7	1.6 ± 0.6	0.9 ± 0.8	1.3 ± 0.7	0.4 ± 0.5	2.6 ± 0.7	8.1 ± 2.9	1.2 ± 0.6	2.2 ± 1.0	24.2 ± 6.2	0.284
	Yes	2.4 ± 0.9	1.7 ± 0.8	2.1 ± 0.7	1.8 ± 0.5	0.8 ± 0.7	1.3 ± 0.7	0.5 ± 0.5	2.7 ± 0.6	8.6 ± 2.4	1.2 ± 0.5	2.3 ± 0.9	25.3 ± 4.9	
Alt. Diet	No	2.3 ± 0.9	1.7 ± 0.9	2.1 ± 0.7	1.7 ± 0.5	0.8 ± 0.7	1.3 ± 0.7	0.4 ± 0.5	2.6 ± 0.7	8.4 ± 2.5	1.2 ± 0.6	2.3 ± 0.9	24.9 ± 5.4	0.247
	Yes	1.8 ± 0.8	2.3 ± 0.8	2.5 ± 0.7	1.7 ± 0.5	0.6 ± 0.7	1.5 ± 0.5	0.6 ± 0.5	2.9 ± 0.3	9.4 ± 2.6	1.2 ± 0.4	2.4 ± 1.0	26.9 ± 5.2	
Total		2.3 ± 0.9	1.7 ± 0.9	2.1 ± 0.7	1.7 ± 0.5	0.8 ± 0.7	1.3 ± 0.7	0.5 ± 0.5	2.6 ± 0.7	8.5 ± 2.5	1.2 ± 0.6	2.3 ± 0.9	24.9 ± 5.4	

Mann–Whitney test (*U*) and Kruskal–Wallis test (*H*) were used with a significance level ≤ 0.05. UW and EO = underweight and extremely obese. N and O = normal and obese.

**Table 5:** Linear regression of nutritional health knowledge score among pregnant women participating in the nutritional health survey, April–May 2022 (*n* = 401).

Predictor	β	SE	CI 95% (Lower–Upper)	t	*Sig.*
Intercept	19.21	1.23	16.80–21.63	15.64	<0.001
Education Level: University vs. Pre-University	3.06	0.55	1.97–4.15	5.53	<0.001
BMI Level: Normal Weight vs. Underweight	2.79	1.17	0.50–5.08	2.40	0.017
BMI Level: Overweight vs. Underweight	3.85	1.29	1.32–6.38	2.99	0.003
BMI Level: Obese vs. Underweight	4.27	1.45	1.42–7.13	2.95	0.003
BMI Level: Extremely Obese vs. Underweight	1.28	1.59	−1.85–4.41	0.80	0.423
City: Prague vs. Pilsen	0.56	0.58	−0.58–1.69	0.96	0.336
Pregnancy Order: First vs. Second or More	0.80	0.52	−0.22–1.82	1.54	0.123
NCD: Yes vs. No	1.13	0.56	0.03–2.23	2.03	0.043

The fit of this model is confirmed by R^2^ value of 0.140.

**Table 6:** Nutritional health literacy of pregnant women participating in the nutritional health survey, April–May 2022 (*n* = 401).

Statement	Response	Pre-University (*n* = 171)	University (*n* = 230)	Total (*n* = 401)	*Sig.*
(Information Acquisition) How difficult was it for you to obtain the necessary information and advice about nutrition during pregnancy?	Very Difficult = 1	0 (0%)	4 (1.8%)	4 (1%)	0.136
	Difficult = 2	11 (6.5%)	26 (11.6%)	37 (9.4%)	
	I do not know = 3	21 (12.5%)	18 (8%)	39 (9.9%)	
	Easy = 4	102 (60.7%)	144 (64%)	246 (62.6%)	
	Very Easy = 5	34 (20.2%)	33 (14.7%)	67 (17%)	
(Information Understanding) How difficult was it for you to understand information from available sources on how to eat properly during pregnancy?	Very Difficult = 1	0 (0%)	5 (2.2%)	5 (1.3%)	0.143
	Difficult = 2	18 (10.8%)	34 (15.2%)	52 (13.3%)	
	I do not know = 3	23 (13.8%)	22 (9.9%)	45 (11.5%)	
	Easy = 4	95 (56.9%)	132 (59.2%)	227 (58.2%)	
	Very Easy = 5	31 (18.6%)	30 (13.5%)	61 (15.6%)	
(Information Appraisal) How difficult was it for you to know which information and advice are really valuable and true?	Very Difficult = 1	6 (3.6%)	15 (6.7%)	21 (5.4%)	0.036
	Difficult = 2	41 (24.6%)	78 (34.7%)	119 (30.4%)	
	I do not know = 3	40 (24%)	39 (17.3%)	79 (20.2%)	
	Easy = 4	66 (39.5%)	76 (33.8%)	142 (36.2%)	
	Very Easy = 5	14 (8.4%)	17 (7.6%)	31 (7.9%)	
(Information Utilisation) How difficult was it for you to use the information and advice you received about nutrition in practice?	Very Difficult = 1	4 (2.4%)	8 (3.6%)	12 (3.1%)	0.031
	Difficult = 2	39 (23.4%)	50 (22.2%)	89 (22.7%)	
	I do not know = 3	44 (26.3%)	20 (8.9%)	64 (16.3%)	
	Easy = 4	68 (40.7%)	129 (57.3%)	197 (50.3%)	
	Very Easy = 5	12 (7.2%)	18 (8%)	30 (7.7%)	
(Professional Help-Seeking) How difficult was it for you to find professional help regarding nutrition during pregnancy?	Very Difficult = 1	0 (0%)	3 (1.3%)	3 (0.8%)	0.036
	Difficult = 2	19 (11.4%)	26 (11.6%)	45 (11.5%)	
	I do not know = 3	46 (27.5%)	80 (35.7%)	126 (32.2%)	
	Easy = 4	76 (45.5%)	94 (42%)	170 (43.5%)	
	Very Easy = 5	26 (15.6%)	21 (9.4%)	47 (12%)	

Mann–Whitney test (*U*) was used with a significance level ≤ 0.05.

**Table 7:** Nutritional health literacy scores of pregnant women participating in the nutritional health survey, April–May 2022 (*n* = 401).

Variable	Outcome	Information Acquisition (1–5)	*Sig.*	Information Understanding (1–5)	*Sig.*	Information Appraisal (1–5)	*Sig.*	Information Utilisation (1–5)	*Sig.*	Professional Help-Seeking (1–5)	*Sig.*	Overall (5–25)	*Sig.*
Age	≤28 yo	4.0 ± 0.8	0.141	3.8 ± 0.9	0.249	3.1 ± 1.1	0.880	3.4 ± 1.0	0.552	3.6 ± 0.9	0.354	18.0 ± 3.7	0.496
	>28 yo	3.8 ± 0.8		3.7 ± 0.9		3.1 ± 1.1		3.4 ± 1.0		3.5 ± 0.9		17.5 ± 3.6	
Education	Pre-Uni	4.0 ± 0.8	0.136	3.8 ± 0.9	0.143	3.3 ± 1.0	0.036	3.3 ± 1.0	0.031	3.7 ± 0.9	0.036	18.0 ± 3.3	0.192
	University	3.8 ± 0.9		3.7 ± 1.0		3.0 ± 1.1		3.4 ± 1.0		3.5 ± 0.9		17.4 ± 3.8	
City	Prague	3.8 ± 0.9	0.067	3.7 ± 1.0	0.044	3.1 ± 1.1	0.117	3.4 ± 1.0	0.418	3.5 ± 0.9	0.524	17.4 ± 3.6	0.249
	Pilsen	4.0 ± 0.8		3.9 ± 0.9		3.2 ± 1.1		3.3 ± 1.0		3.6 ± 0.9		18.0 ± 3.4	
Pregnancy	First	3.7 ± 0.9	0.013	3.6 ± 1.0	0.002	3.0 ± 1.1	0.003	3.3 ± 1.0	0.613	3.5 ± 0.9	0.203	17.1 ± 3.6	0.003
	≥Second	4.0 ± 0.8		3.9 ± 0.8		3.3 ± 1.0		3.4 ± 1.0		3.6 ± 0.9		18.2 ± 3.5	
BMI	<18.5	3.8 ± 0.6	0.657	3.9 ± 0.7	0.465	3.0 ± 0.9	0.542	3.3 ± 0.7	0.906	3.3 ± 0.7	0.319	17.3 ± 2.1	0.637
	18.5–24.9	3.9 ± 0.9		3.8 ± 1.0		3.2 ± 1.1		3.4 ± 1.0		3.6 ± 0.9		17.7 ± 3.9	
	25–29.9	3.8 ± 0.8		3.7 ± 0.9		2.9 ± 1.2		3.3 ± 1.0		3.4 ± 0.8		17.1 ± 3.5	
	30–34.9	3.9 ± 0.9		3.8 ± 0.9		3.3 ± 1.0		3.4 ± 1.1		3.6 ± 0.9		18.0 ± 3.0	
	≥35	3.8 ± 0.8		3.5 ± 1.0		3.1 ± 1.0		3.4 ± 0.8		3.6 ± 0.9		17.3 ± 3.1	
BMI Level	UW and EO	3.8 ± 0.7	0.224	3.7 ± 0.8	0.341	3.1 ± 1.0	0.671	3.4 ± 0.8	0.683	3.5 ± 0.8	0.375	17.3 ± 2.6	0.346
	N and O	3.9 ± 0.9		3.8 ± 0.9		3.1 ± 1.1		3.4 ± 1.0		3.6 ± 0.9		17.6 ± 3.7	
NCD	No	3.9 ± 0.8	0.308	3.7 ± 0.9	0.610	3.1 ± 1.1	0.913	3.4 ± 1.0	0.767	3.6 ± 0.9	0.721	17.7 ± 3.7	0.605
	Yes	3.8 ± 0.9		3.7 ± 0.9		3.1 ± 1.1		3.4 ± 1.0		3.5 ± 0.8		17.5 ± 3.4	
Medicines	No	3.9 ± 0.8	0.616	3.8 ± 0.8	0.492	3.2 ± 1.1	0.573	3.5 ± 1.0	0.049	3.5 ± 0.9	0.979	17.9 ± 3.7	0.438
	Yes	3.9 ± 0.9		3.7 ± 1.0		3.1 ± 1.1		3.3 ± 1.0		3.5 ± 0.9		17.5 ± 3.4	
Alt. Diet	No	3.9 ± 0.8	0.572	3.7 ± 0.9	0.722	3.1 ± 1.1	0.234	3.4 ± 1.0	0.775	3.6 ± 0.9	0.613	17.6 ± 3.6	0.679
	Yes	3.9 ± 1.1		3.7 ± 1.4		3.5 ± 1.3		3.3 ± 1.1		3.4 ± 1.0		17.8 ± 4.7	
Total		3.9 ± 0.8		3.7 ± 0.9		3.1 ± 1.1		3.4 ± 1.0		3.5 ± 0.9		17.6 ± 3.6	

Mann–Whitney test (*U*) and Kruskal–Wallis test (*H*) were used with a significance level ≤ 0.05. UW and EO = underweight and extremely obese. N and O = normal and obese.

## Data Availability

The data that support the findings of this study are available from the corresponding author upon reasonable request.

## Supplementary Materials

The following supporting information can be downloaded at: https://www.mdpi.com/article/10.3390/ijerph20053931/s1, Table S1: Nutritional Health Knowledge Items of Pregnant Women Participating in the Nutritional Health Survey, April–May 2022 (*n* = 401); Table S2. Nutritional Health Knowledge Scores of Pregnant Women Participating in the Nutritional Health Survey, April–May 2022 (*n* = 401); Table S3: Nutritional Health Knowledge Scores of Pregnant Women Participating in the Nutritional Health Survey, April–May 2022, (*n* = 401)
