# Supplementary material for: Nutritional Health Knowledge and Literacy among Pregnant Women in the Czech Republic: Analytical Cross-Sectional Study
Source: Int J Environ Res Public Health. 2023 Feb 22;20(5):3931. doi: 10.3390/ijerph20053931 (PMC10001919; doi:10.3390/ijerph20053931)
Supplement: Supplementary file 1 [file ijerph-20-03931-s001.zip › ijerph-2235418-supplementary.pdf]

Table S1. Nutritional Health Knowledge Items of Pregnant Women Participating in the Nutritional Health Survey, April – May 2022, (*n* = 401)

No.	Question	Answer	Pre-University (*n* = 171)	University (*n* = 230)	Total (*n* = 401)	*Sig.*
1	Which of the following foods is a significant source of iron?	**Meat**	**126 (73.7%)**	**196 (85.2%)**	**322 (80.3%)**	0.006*
		Dairy products	6 (3.5%)	2 (0.9%)	8 (2%)	
		Fruits and vegetables	26 (15.2%)	16 (7%)	42 (10.5%)	
		I do not know	8 (4.7%)	5 (2.2%)	13 (3.2%)	
2	Which of the vitamins supports the absorption of iron in the body?	**Vitamin C**	**38 (22.2%)**	**65 (28.3%)**	**103 (25.7%)**	0.149*
		Vitamin A	21 (12.3%)	24 (10.4%)	45 (11.2%)	
		Vitamin D	32 (18.7%)	56 (24.3%)	88 (21.9%)	
		I do not know	80 (46.8%)	84 (36.5%)	164 (40.9%)	
3	Can a pregnant woman cover the daily need for iron only with a plant-based diet?	Yes	43 (25.1%)	51 (22.2%)	94 (23.4%)	0.683
		**No**	**85 (49.7%)**	**124 (53.9%)**	**209 (52.1%)**	
		I do not know	43 (25.1%)	55 (23.9%)	98 (24.4%)	
4	What is the reason for the increased need for iron in pregnancy?	Optimal growth and development of the child	20 (11.7%)	17 (7.4%)	37 (9.2%)	0.013*
		**Prevention of anaemia in the mother and the developing fetus**	**113 (66.1%)**	**180 (78.3%)**	**293 (73.1%)**	
		Proper development of the child's nervous system	19 (11.1%)	15 (6.5%)	34 (8.5%)	
		I do not know	19 (11.1%)	14 (6.1%)	33 (8.2%)	
5	Which of the following foods is the richest source of folic acid?	Meat	4 (2.3%)	4 (1.7%)	8 (2%)	0.156*
		Dairy products	6 (3.5%)	20 (8.7%)	26 (6.5%)	
		**Fruits and vegetables**	**146 (85.4%)**	**191 (83%)**	**337 (84%)**	
		I do not know	15 (8.8%)	15 (6.5%)	30 (7.5%)	
6	What is the recommended daily amount of folic acid for a pregnant woman?	200 µg	12 (7%)	4 (1.7%)	16 (4%)	<0.001
		**400 µg**	**50 (29.2%)**	**107 (46.5%)**	**157 (39.2%)**	
		600 µg	29 (17%)	38 (16.5%)	67 (16.7%)	
		I do not know	80 (46.8%)	81 (35.2%)	161 (40.1%)	
7	What is the reason for the increased need for folic acid in pregnancy?	Prevention of bone deformities	21 (12.3%)	20 (8.7%)	41 (10.2%)	<0.001*
		**Prevention of cleft neural tube and cleft lip**	**56 (32.7%)**	**118 (51.3%)**	**174 (43.4%)**	
		Growth retardation (slowing down of growth)	38 (22.2%)	55 (23.9%)	93 (23.2%)	
		I do not know	55 (32.2%)	36 (15.7%)	91 (22.7%)	
8	Which foods are an important, well-usable source of calcium for the body?	**Milk and dairy products**	**151 (88.3%)**	**211 (91.7%)**	**362 (90.3%)**	0.667*
		Legumes	10 (5.8%)	11 (4.8%)	21 (5.2%)	
		Egg	5 (2.9%)	3 (1.3%)	8 (2%)	
		I do not know	3 (1.8%)	2 (0.9%)	5 (1.2%)	
9	From which type of vegetable is calcium best usable for the human body?	**Cruciferous vegetables (broccoli, cauliflower, etc)**	**47 (27.5%)**	**93 (40.4%)**	**140 (34.9%)**	0.001*
		Root vegetables (carrots, parsley, celery)	23 (13.5%)	10 (4.3%)	33 (8.2%)	

		Leafy vegetables (lettuce, etc)	24 (14%)	23 (10%)	47 (11.7%)	
		I do not know	76 (44.4%)	104 (45.2%)	180 (44.9%)	
10	Why is it recommended that pregnant women increase their daily calcium intake?	**Proper formation of the child's bones and teeth**	**149 (87.1%)**	**204 (88.7%)**	**353 (88%)**	0.338*
		Development of the child's central nervous system	5 (2.9%)	8 (3.5%)	13 (3.2%)	
		Prevention of genetic defects in the child	1 (0.6%)	5 (2.2%)	6 (1.5%)	
		I do not know	15 (8.8%)	13 (5.7%)	28 (7%)	
11	Which of the listed foods is a significant source of ω-3 unsaturated fatty acids?	**Fish**	**152 (88.9%)**	**221 (96.1%)**	**373 (93%)**	<0.001*
		Dairy products	2 (1.2%)	0 (0%)	2 (0.5%)	
		Egg	3 (1.8%)	0 (0%)	3 (0.7%)	
		I do not know	14 (8.2%)	5 (2.2%)	19 (4.7%)	
12	Why is it recommended to increase the daily intake of ω-3 unsaturated fatty acids for pregnant women?	**For brain development**	**126 (73.7%)**	**185 (80.4%)**	**311 (77.6%)**	0.111*
		Abortion prevention	0 (0%)	3 (1.3%)	3 (0.7%)	
		Prevention of mental retardation	7 (4.1%)	5 (2.2%)	12 (3%)	
		I do not know	38 (22.2%)	37 (16.1%)	75 (18.7%)	
13	Which of the listed foods is a significant source of vitamin D?	**Fish**	**82 (48%)**	**115 (50%)**	**197 (49.1%)**	<0.001*
		Fruit	24 (14%)	7 (3%)	31 (7.7%)	
		Vegetables	29 (17%)	37 (16.1%)	66 (16.5%)	
		I do not know	35 (20.5%)	64 (27.8%)	99 (24.7%)	
14	Why is it recommended to increase pregnant women's daily vitamin D intake?	**Prevention of rickets (child) and bone softening (mother)**	**47 (27.5%)**	**88 (38.3%)**	**135 (33.7%)**	0.006*
		Development of child's vision	13 (%)	27 (%)	40 (%)	
		Development of child's central nervous system	39 (%)	53 (%)	92 (%)	
		I do not know	71 (%)	58 (%)	129 (%)	
15	Which of the following foods is a significant source of iodine?	**Fish and seafood**	**138 (80.7%)**	**206 (89.6%)**	**344 (85.8%)**	0.070*
		Vegetables	1 (0.6%)	0 (0%)	1 (0.2%)	
		Meat	6 (3.5%)	4 (1.7%)	10 (2.5%)	
		I do not know	25 (14.6%)	19 (8.3%)	44 (11%)	
16	Why is it recommended to increase the daily intake of iodine for pregnant women?	**Prevention of psychomotor disorders**	**55 (32.2%)**	**109 (47.4%)**	**164 (40.9%)**	0.013*
		Prevention of visual development disorders	10 (5.8%)	9 (3.9%)	19 (4.7%)	
		Prevention of abortion	12 (7%)	7 (3%)	19 (4.7%)	
		I do not know	92 (53.8%)	104 (45.2%)	196 (48.9%)	
17	Which of the listed foods is a significant source of vitamin A?	**Guts**	**61 (35.7%)**	**118 (51.3%)**	**179 (44.6%)**	0.004*
		Fruits and vegetables	34 (19.9%)	40 (17.4%)	74 (18.5%)	
		Dairy products	11 (6.4%)	6 (2.6%)	17 (4.2%)	
		I do not know	65 (38%)	63 (27.4%)	128 (31.9%)	
18	Which food is a rich source of protein?	**Meat**	**158 (92.4%)**	**214 (93%)**	**372 (92.8%)**	0.078*
		Vegetables	4 (2.3%)	1 (0.4%)	5 (1.2%)	
		Cereals	4 (2.3%)	11 (4.8%)	15 (3.7%)	

		I do not know	5 (2.9%)	2 (0.9%)	7 (1.7%)	
19	Which food is a rich source of carbohydrates?	Meat	9 (5.3%)	12 (5.2%)	21 (5.2%)	0.038*
		**Legumes**	**126 (73.7%)**	**192 (83.5%)**	**318 (79.3%)**	
		Egg	16 (9.4%)	16 (7%)	32 (8%)	
		I do not know	19 (11.1%)	10 (4.3%)	29 (7.2%)	
20	Which food is a rich source of fat?	**Nuts**	**144 (84.2%)**	**223 (97%)**	**367 (91.5%)**	<0.001*
		Rice	1 (0.6%)	1 (0.4%)	2 (0.5%)	
		Banana	13 (7.6%)	1 (0.4%)	14 (3.5%)	
		I do not know	13 (7.6%)	5 (2.2%)	18 (4.5%)	
21	How many servings of fruit (O) and vegetables (Z) should a pregnant woman consume daily?	**2 O + 3 Z**	**61 (35.7%)**	**116 (50.4%)**	**177 (44.1%)**	0.013
		1 O + 3 Z	32 (18.7%)	43 (18.7%)	75 (18.7%)	
		3 O + 2 Z	26 (15.2%)	21 (9.1%)	47 (11.7%)	
		I do not know	52 (30.4%)	50 (21.7%)	102 (25.4%)	
22	Fluid intake is recommended during pregnancy…	1 – 1.5 litre	9 (5.3%)	10 (4.3%)	19 (4.7%)	0.005*
		**1.5 – 3 litres**	**115 (67.3%)**	**184 (80%)**	**299 (74.6%)**	
		> 3 litres	33 (19.3%)	29 (12.6%)	62 (15.5%)	
		I do not know	14 (8.2%)	5 (2.2%)	19 (4.7%)	
23	A pregnant woman should consume fish…	**1 – 2 times a week**	**141 (82.5%)**	**211 (91.7%)**	**352 (87.8%)**	0.009*
		4 – 5 times a week	3 (1.8%)	6 (2.6%)	9 (2.2%)	
		Not at all	6 (3.5%)	3 (1.3%)	9 (2.2%)	
		I do not know	21 (12.3%)	10 (4.3%)	31 (7.7%)	
24	Salt consumption in pregnant women should be…	Increased	6 (3.5%)	6 (2.6%)	12 (3%)	0.012
		**Decreased**	**116 (67.8%)**	**127 (55.2%)**	**243 (60.6%)**	
		Remained the same	29 (17%)	72 (31.3%)	101 (25.2%)	
		I do not know	20 (11.7%)	25 (10.9%)	45 (11.2%)	
25	How many times a day should a pregnant woman eat?	**5 – 7 times a day**	**119 (69.6%)**	**186 (80.9%)**	**305 (76.1%)**	0.008*
		2 – 4 times a day	28 (16.4%)	25 (10.9%)	53 (13.2%)	
		3 times a day	7 (4.1%)	10 (4.3%)	17 (4.2%)	
		I do not know	17 (9.9%)	7 (3%)	24 (6%)	
26	The need for protein is … in the diet of a pregnant woman.	Lower	4 (2.3%)	4 (1.7%)	8 (2%)	0.009*
		The same	43 (25.1%)	39 (17%)	82 (20.4%)	
		**Higher**	**93 (54.4%)**	**162 (70.4%)**	**255 (63.6%)**	
		I do not know	31 (18.1%)	24 (10.4%)	55 (13.7%)	
27	During pregnancy, a woman should have … fibre intake.	Decreased	6 (3.5%)	8 (3.5%)	14 (3.5%)	0.202
		**Increased**	**135 (78.9%)**	**196 (85.2%)**	**331 (82.5%)**	
		I do not know	30 (17.5%)	26 (11.3%)	56 (14%)	
28	Where do we find fibre?	**Fruits and vegetables**	**121 (70.8%)**	**209 (90.9%)**	**330 (82.3%)**	<0.001*
		Milk and dairy products	35 (20.5%)	13 (5.7%)	48 (12%)	

		Meat products	0 (0%)	1 (0.4%)	1 (0.2%)	
		I do not know	15 (8.8%)	7 (3%)	22 (5.5%)	
29	How much kJ -approximately- is the daily energy intake of a pregnant woman increased in the 1st trimester?	**0 – 500 kJ**	**61 (35.7%)**	**121 (52.6%)**	**182 (45.4%)**	0.004*
		1250 kJ	22 (12.9%)	18 (7.8%)	40 (10%)	
		2500 kJ	18 (10.5%)	13 (5.7%)	31 (7.7%)	
		I do not know	69 (40.4%)	78 (33.9%)	147 (36.7%)	
30	How much kJ -approximately- is the daily energy intake of a pregnant woman increased in 2nd and 3rd trimesters?	**1250 kJ**	**66 (38.6%)**	**109 (47.4%)**	**175 (43.6%)**	0.038*
		3500 kJ	20 (11.7%)	30 (13%)	50 (12.5%)	
		4500 kJ	8 (4.7%)	2 (0.9%)	10 (2.5%)	
		I do not know	77 (45%)	89 (38.7%)	166 (41.4%)	
31	Which food do you think corresponds approximately to an energy intake of 1500 kJ?	**150g white yoghurt and 40g muesli**	**32 (18.7%)**	**73 (31.7%)**	**105 (26.2%)**	0.007*
		Tomato sauce with beef and pasta	39 (22.8%)	50 (21.7%)	89 (22.2%)	
		A plate of pure beef broth with vegetables	26 (15.2%)	40 (17.4%)	66 (16.5%)	
		I do not know	73 (42.7%)	67 (29.1%)	140 (34.9%)	
32	Women who are not overweight at the beginning of pregnancy are recommended to gain weight during pregnancy…	10 – 12.5 Kg	90 (52.6%)	117 (50.9%)	207 (51.6%)	0.007*
		**11.4 – 16 Kg**	**46 (26.9%)**	**89 (38.7%)**	**135 (33.7%)**	
		15 – 18 Kg	8 (4.7%)	3 (1.3%)	11 (2.7%)	
		I do not know	27 (15.8%)	20 (8.7%)	47 (11.7%)	
33	Women who are overweight at the beginning of pregnancy are advised to gain weight during pregnancy…	**7 – 11.5 Kg**	**79 (46.2%)**	**106 (46.1%)**	**185 (46.1%)**	0.997
		8 – 10 Kg	43 (25.1%)	57 (24.8%)	100 (24.9%)	
		11.4 – 16 Kg	6 (3.5%)	9 (3.9%)	15 (3.7%)	
		I do not know	43 (25.1%)	58 (25.2%)	101 (25.2%)	
34	Excessive weight gain during pregnancy can negatively affect…	Only maternal health	15 (8.8%)	10 (4.3%)	25 (6.2%)	0.017
		Only fetal health	13 (7.6%)	21 (9.1%)	34 (8.5%)	
		**Maternal and fetal health**	**128 (74.9%)**	**192 (83.5%)**	**320 (79.8%)**	
		I do not know	15 (8.8%)	7 (3%)	22 (5.5%)	
35	When is it recommended for women to start taking folic acid?	**1 month before conception**	**142 (83%)**	**214 (93%)**	**356 (88.8%)**	0.006*
		Only during pregnancy	18 (10.5%)	10 (4.3%)	28 (7%)	
		At the end of pregnancy	1 (0.6%)	0 (0%)	1 (0.2%)	
		I do not know	10 (5.8%)	5 (2.2%)	15 (3.7%)	
36	During pregnancy, a woman should receive vitamin A…	**Increased through diet**	**50 (29.2%)**	**71 (30.9%)**	**121 (30.2%)**	0.004*
		Limited	29 (17%)	69 (30%)	98 (24.4%)	
		As food supplements	20 (11.7%)	14 (6.1%)	34 (8.5%)	
		I do not know	70 (40.9%)	76 (33%)	146 (36.4%)	
37	Which of the listed fish has a higher mercury content?	**Pike**	**20 (11.7%)**	**45 (19.6%)**	**65 (16.2%)**	0.037
		Salmon	45 (26.3%)	55 (23.9%)	100 (24.9%)	
		Cod	49 (28.7%)	77 (33.5%)	126 (31.4%)	
		I do not know	57 (33.3%)	53 (23%)	110 (27.4%)	

38	*Listeria monocytogenes* is a bacterium that a pregnant woman can become infected with when eating…	**Soft-ripened cheese with fungal cultures**	**125 (73.1%)**	**168 (73%)**	**293 (73.1%)**	0.431*
		Canned meat	3 (1.8%)	7 (3%)	10 (2.5%)	
		Contaminated water	9 (5.3%)	16 (7%)	25 (6.2%)	
		I do not know	34 (19.9%)	36 (15.7%)	70 (17.5%)	
39	*Salmonella* is a bacterium that a pregnant woman can become infected with, especially when eating…	Improper washing of fruits and vegetables	9 (5.3%)	11 (4.8%)	20 (5%)	0.437*
		**Undercooked meat and eggs**	**155 (90.6%)**	**216 (93.9%)**	**371 (92.5%)**	
		When reheating food	1 (0.6%)	0 (0%)	1 (0.2%)	
		I do not know	4 (2.3%)	2 (0.9%)	6 (1.5%)	
40	*Listeria* mainly poses a risk during pregnancy…	Food poisoning	28 (16.4%)	41 (17.8%)	69 (17.2%)	0.900*
		**Miscarriage/stillbirth**	**75 (43.9%)**	**100 (43.5%)**	**175 (43.6%)**	
		Excruciating pain	5 (2.9%)	10 (4.3%)	15 (3.7%)	
		I do not know	63 (36.8%)	78 (33.9%)	141 (35.2%)	

Chi-squared test (χ^2) and Fisher's exact test (*) were used with a significance level ≤ 0.05. The correct answers were highlighted in bold font.

Table S2. Nutritional Health Misconceptions Scores of Pregnant Women Participating in the Nutritional Health Survey, April – May 2022, (n = 401)

Variable	Outcome	Misconception I (1 – 5)	*Sig.*	Misconception II (1 – 5)	*Sig.*	Misconception III (1 – 5)	*Sig.*	Misconception IV (1 – 5)	*Sig.*	Misconception V (1 – 5)	*Sig.*
**Age**	≤ 28 yo	1.5 ± 0.7	0.748	1.8 ± 1.0	0.312	3.3 ± 1.2	**<0.001**	2.6 ± 1.2	0.887	3.3 ± 1.1	**0.017**
	> 28 yo	1.5 ± 0.8		1.6 ± 0.7		2.8 ± 1.2		2.6 ± 1.2		3.6 ± 1.1	
**Education**	Pre-Uni	1.5 ± 0.6	0.726	1.8 ± 1.0	**0.002**	3.2 ± 1.2	**<0.001**	2.8 ± 1.2	**<0.001**	3.7 ± 1.0	**0.024**
	University	1.6 ± 0.8		1.5 ± 0.6		2.7 ± 1.2		2.4 ± 1.1		3.4 ± 1.1	
**City**	Prague	1.6 ± 0.8	0.296	1.6 ± 0.7	0.431	2.8 ± 1.2	**0.022**	2.6 ± 1.1	0.928	3.5 ± 1.1	0.779
	Plzen	1.4 ± 0.6		1.7 ± 1.0		3.1 ± 1.3		2.6 ± 1.2		3.5 ± 1.1	
**Pregnancy**	First	1.6 ± 0.8	0.757	1.6 ± 0.8	0.253	2.9 ± 1.2	0.615	2.6 ± 1.2	0.812	3.4 ± 1.0	**0.041**
	≥ Second	1.5 ± 0.7		1.7 ± 0.8		2.9 ± 1.2		2.6 ± 1.2		3.6 ± 1.1	
**Trimester**	2nd Trim	1.3 ± 0.6	0.726	1.3 ± 0.6	0.539	2.7 ± 1.2	0.738	2.7 ± 0.6	0.779	3.7 ± 0.6	0.983
	3rd Trim	1.5 ± 0.8		1.6 ± 0.8		2.9 ± 1.2		2.6 ± 1.2		3.5 ± 1.1	
**BMI**	< 18.5	1.6 ± 1.1	0.261	1.9 ± 1.2	0.935	3.0 ± 1.2	0.894	2.4 ± 1.2	0.454	3.0 ± 1.2	0.121
	18.5 – 24.9	1.5 ± 0.7		1.6 ± 0.8		2.9 ± 1.2		2.6 ± 1.2		3.5 ± 1.1	
	25 – 29.9	1.5 ± 0.8		1.6 ± 0.8		2.9 ± 1.2		2.6 ± 1.0		3.7 ± 1.1	
	30 – 34.9	1.9 ± 1.1		1.7 ± 0.8		2.8 ± 1.2		2.6 ± 1.2		3.6 ± 0.9	
	≥ 35	1.4 ± 0.5		1.8 ± 1.1		3.1 ± 1.4		3.0 ± 1.1		3.7 ± 0.9	
**BMI Level**	UW & EO	1.5 ± 0.8	0.447	1.8 ± 1.1	0.539	3.1 ± 1.3	0.439	2.7 ± 1.2	0.415	3.3 ± 1.1	0.187
	N & O	1.5 ± 0.8		1.6 ± 0.8		2.9 ± 1.2		2.6 ± 1.2		3.6 ± 1.1	
**NCD**	No	1.5 ± 0.8	0.442	1.7 ± 0.8	**0.050**	3.0 ± 1.2	0.118	2.6 ± 1.2	0.343	3.5 ± 1.1	0.333
	Yes	1.5 ± 0.8		1.5 ± 0.7		2.8 ± 1.2		2.5 ± 1.0		3.6 ± 1.1	
**Medicines**	No	1.6 ± 0.8	0.802	1.7 ± 0.9	0.148	3.1 ± 1.2	0.042	2.7 ± 1.3	0.350	3.4 ± 1.2	0.098
	Yes	1.5 ± 0.8		1.6 ± 0.7		2.8 ± 1.2		2.5 ± 1.1		3.6 ± 1.0	
**Alt. Diet**	No	1.5 ± 0.8	0.396	1.6 ± 0.8	0.297	2.9 ± 1.2	0.500	2.6 ± 1.2	0.835	3.5 ± 1.1	0.737
	Yes	1.3 ± 0.5		1.4 ± 0.7		3.2 ± 1.4		2.6 ± 1.4		3.7 ± 0.9	
**Total**		1.5 ± 0.8		1.6 ± 0.8		2.9 ± 1.2		2.6 ± 1.2		3.5 ± 1.1	

Mann-Whitney test (U) and Kruskal-Wallis test (H) were used with a significance level ≤ 0.05. UW & EO = Underweight and Extremely Obese. N & O = Normal and Obese.

Table S3. Nutritional Health Knowledge Scores of Pregnant Women Participating in the Nutritional Health Survey, April – May 2022, (*n* = 401)

Number of points obtained	Number of participants	Number of respondetns relatively
4	1	0,25%
6	2	0,50%
8	2	0,50%
10	1	0,25%
11	2	0,50%
12	2	0,50%
13	4	1,00%
14	2	0,50%
15	7	1,75%
16	8	2,00%
17	3	0,75%
18	10	2,49%
19	12	2,99%
20	24	5,99%
21	16	3,99%
22	17	4,24%
23	20	4,99%
24	30	7,48%
25	34	8,48%
26	35	8,73%
27	32	7,98%
28	34	8,48%
29	31	7,73%
30	25	6,23%
31	15	3,74%
32	13	3,24%
33	10	2,49%
